# *Mycobacterium ulcerans* Disease with Unusual Sites Not to Be Ignored

**DOI:** 10.1155/2014/639374

**Published:** 2014-10-16

**Authors:** Sangaré Abdoulaye, Kourouma Sarah Hamdan, Kouassi Yao Isidore, Ecra Elidjé Joseph, Kaloga Mamadou, Gbery Ildevert Patrice

**Affiliations:** Department of Dermatology of the University Teaching Hospital of Treichville, Riviera, BP 408 Cidex 03 Abidjan, Cote d'Ivoire

## Abstract

*Objective*. The usual preferential site of BU is in the limbs. In our experience, we noticed atypical and often misleading sites which pose serious issues for the diagnosis and often for the treatment. *Methods*. This is a retrospective study conducted over a period of ten years of BU treatment at the Department of Dermatology of the University Teaching Hospital of Treichville (Abidjan, Côte d'Ivoire). We included in this study all BU cases with atypical site diagnosed clinically and confirmed either by the histology, by smear, or by PCR. *Results*. Epidemiologically, the age of patients ranged from 3 to 72 years with a median age of 14.2 years. Children aged less than 15 years were affected in almost 80% of case. The clinical table was dominated by ulcerated forms in 82.1% of cases. The unusual topography mostly observed was that of the torso (thorax, back, and abdomen) in 76.8% of cases. *Conclusion*. BU is an endemic disease in Côte d'Ivoire where it constitutes a serious public health issue. Several years following its first cases, BU still is little known. This dermatosis may present atypical misleading clinical aspects which must be ignored.

## 1. Introduction

*Mycobacterium ulcerans* (MU) also known as Buruli ulcer (BU) named after the District of Uganda where an epidemic occurred in the 1960s is mycobacteriosis [1]. This disease believed to be mysterious by many parents is characterized by preulcerative lesions leading in the long term to major chronic cutaneous deterioration often associated to definitive disabilities [2]. In Côte d'Ivoire, Buruli ulcer which is the second mycobacteriosis after tuberculosis constitutes an emerging endemic. This is the reason why the government initiated, since 1998, the National Programme of Fight against Mycobacterium Ulcers (PNUM) in Côte d'Ivoire.

Its preferential site in 9 out of 10 cases is in lower limbs [3, 4]. However, in our experience, we observed some unusual sites. So the purpose of this study is to contribute to a better understanding of them.

The specific objectives of this study are to determine sociodemographic characteristics and to describe clinical and topographical aspects of such unusual sites.

## 2. Patients and Method

This is a retrospective, cross-sectional, and descriptive study related to BU cases observed over a period of ten years (i.e., from 2003 to 2013). This study was conducted in the Dermatology Department of the University Teaching Hospital of Treichville which is the reference centre for cutaneous pathologies in Abidjan and served as the head office of the PNUM.

We included patients, irrespective of their gender and age, who over the study period developed an unusual (atypical) ulcer or a nodule clinically evoking* Mycobacterium ulcerans.*

We considered, as the usual or typical site of BU, any ulcer that is found on the limbs and more specifically on lower limbs.

However, any site, other than the limb, is said to be unusual, atypical, or misleading. The subject matter of this study is unusual sites.

The BU was diagnosed on the basis of clinical and paraclinical arguments.

With regard to clinical aspects, we considered the existence of the following:

(i) manifestations which evoke the inception of a BU: nodule, oedema, and infiltrated plate,
(ii) at latter stage, the characteristic ulceration with its thickened, devitalized, and peeled edges, surpassing the base.

With regard to paraclinical aspects, there should be at least the result of one of the following examinations:

- the histology of a nodule, an oedema, or an infiltrated plate with Ziehl-Neelsen stain;
- the smear conducted from the exudates of the ulceration edges with Ziehl-Neelsen stain;
- the PCR* (polymerase chain reaction) conducted on the exudate.*

Cutaneous biopsies were conducted at the Department of Dermatology and plates were read in the anatomic pathology laboratory of the same University Teaching Hospital. The smear and PCR were conducted by the “*Institut Pasteur of Côte d'Ivoire.*”

The histology was revelatory of a BU case if an infiltrate of lymphocyte, histiocytosis, and hypodermic necrosis were found or if AFB (acid-alcohol-fast Bacilli) were revealed by the Ziehl-Neelsen stain method.

With regard to smear, a positive Ziehl-Neelsen stain was considered as a potential BU case. However, when the Ziehl-Neelsen stain was negative, a PCR* (polymerase chain reaction) *was conducted on the sampling in order to confirm the diagnosis.

The smear and histology are less expensive but they have an average sensibility. Moreover, such examinations have a poor specificity and do not permit discriminating mycobacteria.

With regard to PCR, its sensibility and specificity are above 90%.

On the basis of clinical and paraclinical arguments, we collected in all 213 BU records comprising classic sites as well as unusual sites.

We did not include in this study all the incomplete records which had no paraclinical data.

## 3. Results

### 3.1. Overall Incidence of BU during the Study Period

During the study period, we recorded in the whole department 42495 patients who came for consultation for various dermatosis. Of the whole population who came for consultation in our department over the study period, we observed 213 BU cases, that is, an overall incidence of 0.5%.

### 3.2. Sociodemographic Characteristics of Atypical BU (Table 1)

#### 3.2.1. Incidence of BU with Atypical Site

Of the 213 cases of BU collected, we observed 39 cases of BU with atypical site (i.e., 18.3%) and 174 cases of BU found on the limbs (81.6%).

#### 3.2.2. Age of Patients with BU of Atypical Site

The age of patients ranged from 3 to 72 years. The mean age was 14.2 years. Children aged less than 15 years were affected in almost 80% of the cases.

#### 3.2.3. Gender of Patients with BU of Atypical Site

We observed a female predominance of 71.7%. The sex ratio was 2.5.

### 3.3. Clinical Characteristics of BU Cases with Atypical Site

Clinical forms of atypical site were dominated by ulcerated forms (82.1%).

### 3.4. Topographical Aspects of BU with Atypical Site

Sites on the torso (thorax, abdomen, and back) were the most frequent forms (76.8%).

## 4. Discussion

BU is a mycobacteriosis which rages under the form of endemic foci in our country to the extent that, in 1995, the Ivorian government set up a National Programme of Fight against Mycobacterium Ulcers (PNUM). Unlike its usual sites in the limbs which are well documented, atypical sites are not. As a matter of fact, they are misleading forms whose diagnosis and treatment are difficult and should not be ignored by practitioners; they are likely to threaten the functional prognosis and survival in some cases. Such forms in our study had a hospital incidence of 18.3%.

Sociodemographic characteristics of misleading forms are similar to usual forms of BU. BU with atypical sites affects, like its classic form, mostly children. In 79.5% of the cases, atypical forms were observed in children aged less than 15 years. The BU predominance in this target is observed in various studies [5–7]. It was related to a deficit of immunity in those children [8]. The factor accounting for that situation is the absence of specific vaccine protection against MU and the antituberculosis vaccination, BCG (Bacilli Calmette-Guerin), offers only a transitional protection which subsides from 6 months to 1 year [9, 10]. Moreover, games or fishing, by those children near waters, exposes them to cutaneous microtrauma which favours the penetration of MU in the body [11].

In our study, females patients were the most affected people. The epidemiological profile classically shows that the BU affects the children without distinction of sex. This ascendancy of females in this study would be of recruitment bias.

They represent 71.7% of patients. As a matter of fact, women, in our traditions, are in charge of household chores which are mainly laundry and dishes. These chores are also conducted near stretch of water and swamps in 77% of the cases (please refer to Table 1).

Our country, Côte d'Ivoire, is a country with limited resources. The minimum wage is $120. Due to poverty, only few households have access to drinking water. As a result, many families are obliged to use swamp water for the needs of their household. Though the BU transmission mode is not clearly identified, one knows that contact with those stagnant waters is a major factor in the outbreak of the disease [12]. As a matter of fact, a PCR conducted enabled us to discover freshwater bugs of the like of* Naucoris* and* Diplonychus* on the roots of some aquatic plants which might shelter MU [1].

In our experience, patients are barely consulted at the inception which is oedema (10.2%) and nodule (7.7%). However, when the disease is diagnosed at this stage, the treatment is less complex and the prognosis is better [13]. However, in 82.1% of the cases, patients go to hospital at the ulcerative stage which is the severest form, the most dilapidating, with at times a risk of incapacitating scares in children [14]. This negligence of diseases can be explained by poverty. As a matter of fact, due to economic reasons, those patients undertake self-medication at the inception of the pathology. They would only go to health centres, after several weeks or months when their treatments have failed or when the case has developed into some complications. As well, those unusual sites of BU are sometimes very misleading and give rise to misdiagnosis and delays in the efficient treatment, given that it is ignored by many practitioners. This is the reason of our vehement advice to our colleagues in endemic zones to undertake in case of doubt the incisional biopsy of any nodule in order to conduct histological examination and, at the ulceration stage, conduct wound edge swabbing in view of conducting a PCR which would allow for early diagnosis of BU within 48 hours [15, 16].

Histology and smear are examinations with an average sensibility and a poor specificity. The poor performance of these examinations could actually induce a bias of recruitment by omitting confirmed cases of BU or registering false cases. However given that these tests are less expensive and easy to carry out, they permit defining probable cases of BU in endemic zones like ours, according to the WHO [17].

However, the PCR has quite a good sensibility and its specificity is above 90%. In the event of a positive result, it allows the confirmation of BU cases [17]. But its high cost prevents its use as a routine examination.

**Figure 1:**
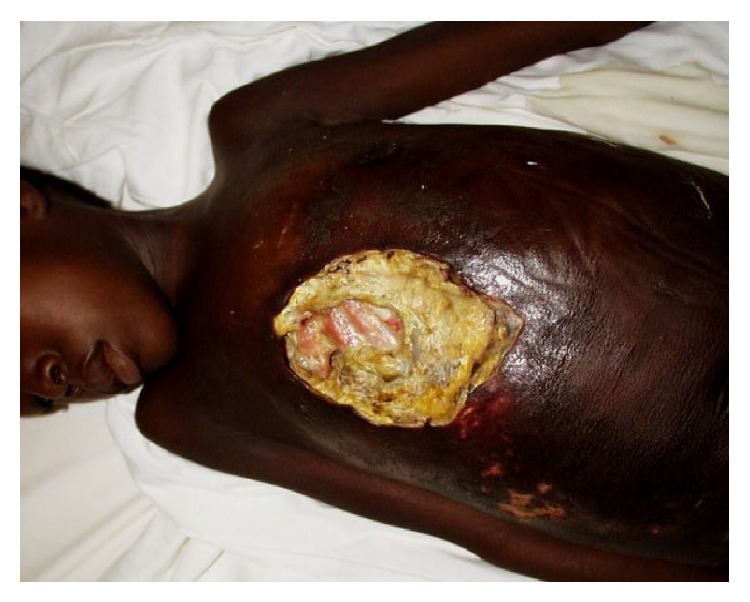
Thoracic BU revealing the ribs of an 8-year-old girl.

**Figure 2:**
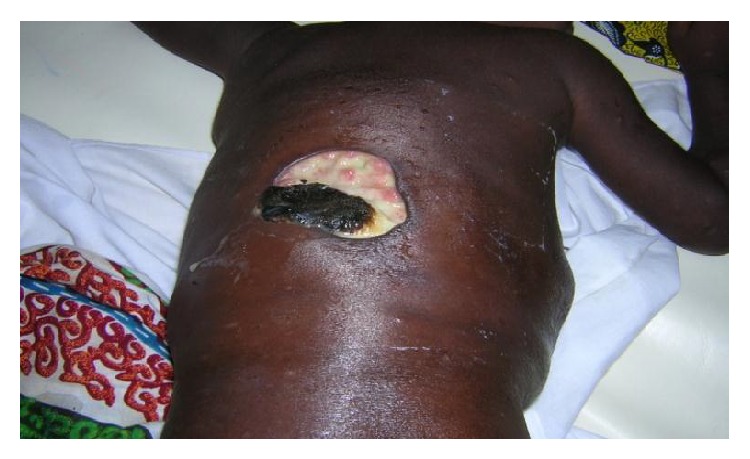
BU of the back.

With regard to topography, BU may affect any part of the human body but limbs remain its predilection site [18–20]. Unusual topographic aspects observed were predominantly in the torso (thorax, abdomen, and back) in 76.8% (Figures 1 and 2). There are severe forms which could threaten survival due to pneumothorax type complications or pleurisy which go along with them in some cases [21].

**Figure 3:**
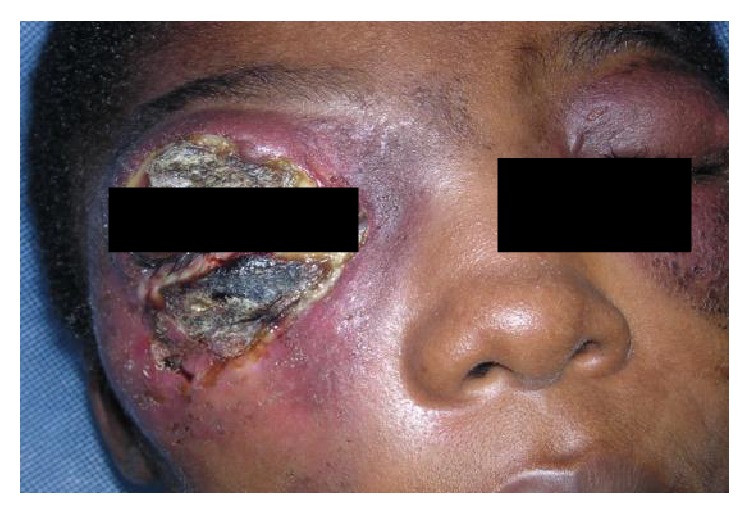
Facial BU above and beneath right palpebral fissure.

Apart from these predominant forms found on the torso, the study revealed moreover facial affections of up to 15.3% (Figure 3). These forms located on the face, in addition to presenting diagnostic difficulties, pose a problem with regard to their surgical treatment given their proximity to the eyes. The functional prognostic of these forms is related to the likeliness of extension of BU to the eyes as it was observed with some patients.

## 5. Conclusion

BU is an endemic disease in Côte d'Ivoire where it constitutes a serious public health issue. Several years following its first description by Mac Callum, BU remains understudied. As a matter of fact, in addition to its mode of transmission which is yet to be elucidated, this dermatosis may clinically present atypical and misleading aspects likely to threaten survival. Future researches could help for a better understanding of the various unknown aspects of this disease.

## Figures and Tables

**Table 1:** Epidemiological and clinical characteristics of atypical forms.

Parameters	Numbers (*N*)	Percentage (%)
Incidences of atypical site		
Sites on the limbs	174	81.6
Atypical sites	**39**	**18.3**
Sociodemographic characteristics		
Age		
< or = 15 years	**31**	**79.5**
>15	08	20.5
Sex		
Female	**28**	**71.7**
Male	11	28.3
Residence		
Swampy zones	**30**	**77**
Far	09	23
Clinical aspects		
Ulcerated forms	**32**	**82.1**
Edematous forms	04	10.2
Nodular forms	03	7.7
Topographic aspects		
Thorax	**12**	**30.7**
Abdomen	**10**	**25.6**
Back	**08**	**20.5**
Face	06	15.3
Genitals	03	7.7

## Abbreviations

AFB: Acid-alcohol-fast Bacilli
BCG: Bacilli Calmette-Guerin
MU: *Mycobacterium ulcerans*
PCR: Polymerase chain reaction
PNUM: National Programme of Fight against Mycobacterium Ulcers
B.U: Buruli ulcers.
